# Effects of Angiotensin-(1-7) and Angiotensin II on Acetylcholine-Induced Vascular Relaxation in Spontaneously Hypertensive Rats

**DOI:** 10.1155/2019/6512485

**Published:** 2019-11-20

**Authors:** Feng Zhang, Yu Xu, Yan Pan, Shuo Sun, Aidong Chen, Peng Li, Changlei Bao, Jian Wang, Haiyang Tang, Ying Han

**Affiliations:** ^1^Key Laboratory of Targeted Intervention of Cardiovascular Disease, Collaborative Innovation Center of Translational Medicine for Cardiovascular Disease, Department of Physiology, Nanjing Medical University, Nanjing, Jiangsu 211166, China; ^2^Department of Geriatrics, Shandong Provincial Hospital Affiliated to Shandong First Medical University, Jinan, Shandong 250021, China; ^3^Department of Cardiology, The First Affiliated Hospital of Nanjing Medical University, Nanjing, Jiangsu 210029, China; ^4^College of Veterinary Medicine, Northwest A&F University, Yangling, Shaanxi 712100, China; ^5^State Key Laboratory of Respiratory Disease, National Clinical Research Center for Respiratory Disease, Guangzhou Institute of Respiratory Health, The First Affiliated Hospital of Guangzhou Medical University, Guangzhou, Guangdong 510120, China

## Abstract

Endothelial dysfunction of small arteries occurs in patients with hypertension and in various hypertensive models. Endothelial function is usually evaluated by the degree of acetylcholine- (ACh-) induced vascular relaxation. Our previous study has found that compared to Wistar-Kyoto rats (WKY), ACh-induced vasodilatation was attenuated significantly in the mesenteric artery (MA), coronary artery (CA), and pulmonary artery (PA) of spontaneously hypertensive rats (SHR). This study investigated the influence of angiotensin- (Ang-) (1-7) and Ang II on blood pressure and ACh-induced vascular relaxation, as well as their interactive roles and downstream signal pathways in SHR and WKY. Intravenous injection of Ang II significantly increased, while Ang-(1-7) decreased the mean arterial pressure (MAP) in SHR. Ang-(1-7) improved ACh-induced relaxation in the MA, CA, and PA of SHR, while Ang II further attenuated it, which were inhibited by pretreatment with Mas receptor antagonist A-779 or AT_1_ receptor antagonist losartan, respectively. Ang-(1-7) decreased the basal arterial tension, and Ang II induced great vasoconstriction in SHR. Pretreatment with Ang-(1-7) inhibited the Ang II-induced pressor response, vasoconstriction, and the effects on ACh-induced relaxation in SHR. AT_1_ receptor expression was higher, while nitric oxide (NO), cGMP, and protein kinase G (PKG) levels of arteries were lower in SHR than in WKY. Ang II decreased, while Ang-(1-7) increased, the levels of NO, cGMP, and PKG of arteries. In addition, pretreatment with Ang-(1-7) inhibited the Ang II-induced reduction of NO, cGMP, and PKG in SHR. These results indicate that the activation of the Mas receptor by Ang-(1-7) can improve endothelial function and decrease MAP in SHR and inhibit the deteriorative effect of Ang II on endothelial function through the NO-cGMP-PKG pathway.

## 1. Introduction

The integrity of vascular endothelial function plays an important role in maintaining equilibrium and homeostasis of vascular tension in a normal physiological state [1]. Vascular endothelial cells (VECs) release vasoconstrictor factors, such as endothelin-1. More importantly, VECs can also release several vasodilators, such as nitric oxide (NO), endothelium-derived hyperpolarizing factor (EDHF), and prostaglandin (PGI_2_). Among them, NO plays the most important roles in vasodilatation [2]. Endothelial nitric oxide synthase (eNOS) catalyzes the release of NO, which then diffuses into adjacent vascular smooth muscle cells (VSMCs), resulting in relaxation of smooth muscle through activation of the intracellular guanylate cyclase- (GC-) cGMP-protein kinase G (PKG) signal pathway [3]. Acetylcholine (ACh) stimulates endothelial cells to release NO and induce vasodilatation; therefore, ACh-induced vasodilatation is usually used to evaluate endothelial function [1, 4].

Some factors, such as hypoxia, increased oxidative stress, increased inflammatory factors, and vascular mechanical dystonia, hurt artery endothelial cells and cause endothelial dysfunction [5]. The major cause of endothelial dysfunction is the imbalance of the release of vasoconstrictor factors and vasorelaxation factors, resulting in the decline of vascular diastolic function and the enhancement of vascular contraction function [6, 7]. Patients with essential hypertension [8, 9] or secondary hypertension [10, 11] have been reported to show endothelial dysfunction in small arteries. This phenotype is also typical in multiple hypertensive models [12]. Impaired NO release caused by endothelial dysfunction of small arteries and subsequent impaired vascular relaxation are implicated in the development and progression of hypertension [13] and involved in the further progression of organ damage in hypertension [14]. Our previous study has also found that compared to Wistar-Kyoto rats (WKY), ACh-induced vasodilatation was attenuated significantly in the mesenteric artery (MA), coronary artery (CA), and pulmonary artery (PA) of spontaneously hypertensive rats (SHR) [15], but the mechanisms involved in it are still not very clear.

Biologically active peptide angiotensin- (Ang-) (1-7) is one important member of the renin-angiotensin system (RAS) family which regulates cardiovascular activity by activation of the Mas receptors [16–19]. The Mas receptor is massively expressed in the VECs and selectively blocked by its specific antagonist D-alanine-Ang-(1-7) (A-779) [20–22]. It has been reported that Ang-(1-7) in a peripheral circulation system prevents the development of chronic hypertension and end-organ damage in SHR [19, 23, 24], but the specific mechanism for this antihypertensive effect of Ang-(1-7) is still unknown. A study has shown that Ang-(1-7) can improve endothelial function and delays the development of cardiac remodeling and heart failure in rats with myocardial infarction [25]. Ang-(1-7) induces the mesenteric arterial relaxation of normal rats [26, 27], and intravenous injection of Ang-(1-7) decreases blood pressure [28]. In addition, some studies have shown that the Ang-(1-7)/Mas receptor axis plays opposite roles to Ang II and acts as a counterregulator of the classic Ang II/AT_1_-mediated effects in some peripheral tissues [29–32]. Activation of the Mas receptor blocks Ang II-induced vasoconstriction, and interfering with the expression of the Mas receptor significantly enhances the effect of Ang II on vascular tension [33]. Ang-(1-7) plays an antagonistic role in an Ang II-induced pressor effect [34] and inhibits Ang II-induced phosphorylation of ERK, p38, and JNK [35]. Ang-(1-7) inhibits myocardial hypertrophy induced by Ang II by activating Mas receptors [36]. Given the current body of data, Ang-(1-7) is a relevant area of study for mitigating negative endothelial functions and involved roles of Ang II. Specific effects of Ang-(1-7) and Ang II on endothelial dysfunction and the interaction between Ang-(1-7) and Ang II in hypertension are still unclear and warrant further investigation.

In this study, we used the MA, CA, and PA to determine the effects of Ang-(1-7) and Ang II on ACh-induced arterial relaxation, the influence of Ang-(1-7) on Ang II-induced responses, and their signal molecular mechanisms in SHR and WKY.

## 2. Materials and Methods

All procedures were approved by Nanjing Medical University Experimental Animal Care and complied with the Guide for the Care and Use of Laboratory Animals published by the US National Institutes of Health (NIH publication, 8th edition, 2011). Thirteen-week-old male WKY and SHR rats (Vital River Laboratory Animal Technology Co. Ltd., Beijing, China) were kept in a temperature-controlled room on a 12 h-12 h light-dark cycle with free access to standard chow and water. The major methods used in this study were done as described in a previous report [15].

### 2.1. Intravenous Injection

Intravenous injection of 100 *μ*L of either Ang-(1-7) or Ang II was performed through an external jugular vein catheter; injection was performed at a controlled rate for 10 minutes. A dual-channel microdialysis infusion syringe pump (53101V, Stoelting Co., Illinois, USA) was used to control the injection rate.

### 2.2. Mean Arterial Pressure (MAP) and Heart Rate (HR) Recording

Rats were anesthetized with urethane (800 mg/kg, intraperitoneal). A cannula was inserted into the right carotid artery and connected to a pressure transducer (MLT0380, ADInstruments, Australia). Arterial blood pressure, MAP, and HR were measured continuously.

### 2.3. Systolic Blood Pressure (SBP) Measurements

The SBP of the tail artery of conscious rats was measured with a noninvasive computerized tail-cuff system (NIBP, ADInstruments, Australia) as indicated in our previous report [37]. The SBP was obtained by averaging 10 measurements.

### 2.4. Isometric Tension Measurements in Arteries

In order to evaluate vascular function, the isometric tension of arteries was measured as previously described [15, 38]. Arterial rings (1.0 to 1.2 mm in length) from the third-order coronary arteries, pulmonary arteries, or mesenteric arteries were isolated from rats. The tissue was mounted onto four-chambered myographs (620M, DMT, Denmark) and set at a resting tension of 0.1 g. In order to evaluate the functionality of each arterial ring, the tissue was contracted by a high K^+^ solution (as previously reported [15]). Then, prostaglandin F2*α* (PGF 2*α*) was used to induce arterial ring contraction, followed by 6 doses of ACh (10^−9^~10^−4^ mol/L) administrated in a dose-dependent manner to induce vasodilatation. The degree of relaxation is shown as a percentage of PGF 2*α*-induced contraction. To determine the effects of Ang-(1-7) or Ang II on ACh-induced vasodilatation, the chemicals were added 20 minutes before the contraction induced by PGF 2*α*.

### 2.5. Artery Sample Preparation

The MA, CA, or PA were isolated from rats and were flash-frozen in liquid nitrogen and stored at -70°C. Arterial tissues were homogenized and centrifuged; the supernatant was collected to extract total protein. Total protein concentration was measured using a commercial protein assay kit (BCA, Pierce Chemical, USA). The protein was then used for the detection of AT_1_ receptor protein expression by western blotting or immunohistochemistry. To determine the effects of Ang-(1-7) or Ang II on NO, cGMP, and PKG levels of arteries, the MA, CA, or PA were isolated from rats and incubated in Krebs-Henseleit solution (as previously described [15]) in combination with Ang-(1-7) or Ang II for 20 minutes. The arteries were then flash-frozen in liquid nitrogen and stored at -70°C.

### 2.6. Western Blotting

The AT_1_ receptor protein expressions in the arteries were determined by western blotting as described in our previous reports [39, 40]. Briefly, proteins purified from the supernatant of artery tissue homogenates were transferred to a nitrocellulose membrane after being run on a gel. An antibody specific for the AT_1_ receptor (diluted 1 : 500; Abcam, Cambridge, MA, USA) was used, followed by blotting with horseradish peroxidase-conjugated goat anti-rabbit IgG (diluted 1 : 5000; Immunology Consultants Lab, Portland, OR, USA). An enhanced chemiluminescence ECL system (Pierce Chemical, Rockford, IL, USA) was used to visualize the blots. Protein loading was normalized by probing blots with the house-keeping gene, glyceraldehyde-3-phosphate dehydrogenase (GAPDH) (1 : 5000; Bioworld Technology Inc., Louis, MN, USA); the total protein amount of the AT_1_ receptor was derived after normalization to GAPDH.

### 2.7. Measurement of NO, cGMP, and PKG Levels

NO production of arteries was detected by a Nitrate/Nitrite Colorimetric Assay Kit (Cayman Chemical Co., Ann Arbor, MI, USA), to measure the concentration of stable nitrate and nitrite metabolites. In order to quantify the levels of cGMP and PKG in the arteries, a commercially available enzyme immunoassay kit was used (Cayman Chemical Co., Ann Arbor, MI, USA; Yi Fei Xue Biotechnology, Nanjing, China).

### 2.8. Immunohistochemistry

AT_1_ receptor immunohistochemistry of the arteries was performed with a commercially available immunohistochemistry kit (Abcam, MA, USA). Coronal sections (5 *μ*m) were made from the isolated arteries and incubated overnight at 4°C with an antibody specific for the AT_1_ receptor (diluted 1 : 500; Abcam, Cambridge, MA, USA) protein. The arteries were then incubated with a secondary antibody (biotinylated goat anti-rabbit IgG) for one hour, followed by staining with DAB in accordance with the manufacturer's instructions. AT_1_ receptor immunoreactivity was observed using light microscopy (DP70, Olympus, Tokyo, Japan) after covering the section slides with mounting media.

### 2.9. Chemicals

All chemicals were dissolved in normal saline to create working solutions at stated concentrations. Ang-(1-7) and D-alanine-Ang-(1-7) (A-779, an antagonist of Mas receptors) were obtained from Bachem (Bubendorf, Switzerland). Ang II, losartan, prostaglandin F2*α* (PGF 2*α*), and acetylcholine (ACh) were purchased from Sigma Chemical Co. (St. Louis, MO, USA).

### 2.10. Statistical Analysis

Data are expressed as the mean ± SE. One-way or two-way ANOVA was used, followed by the Bonferroni test, for post hoc analysis when multiple comparisons were made. *P* < 0.05 was considered statistically significant.

## 3. Results

### 3.1. Effects of Ang-(1-7) and Ang II on Blood Pressure and Heart Rate

**Figure 1:**
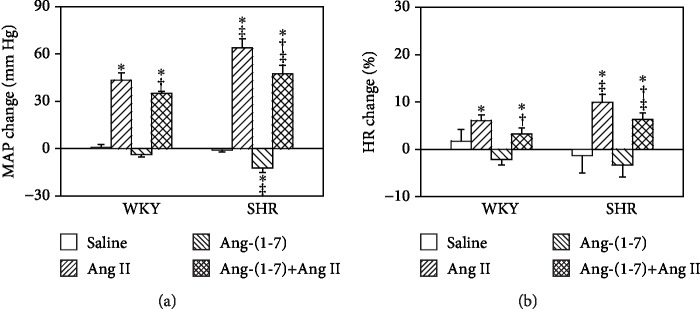
Effects of intravenous injection of saline, Ang II (10^−4^ mol/L), Ang-(1-7) (10^−4^ mol/L), and Ang-(1-7)+Ang II on mean arterial pressure (MAP) and heart rate (HR) in WKY and SHR. Values are the mean ± SE. ^∗^*P* < 0.05 compared with saline. ^†^*P* < 0.05 compared with Ang II alone. ^‡^*P* < 0.05 compared with WKY. *n* = 6 for each group.

Intravenous injection of Ang II significantly increased MAP (Figure 1(a)) and HR (Figure 1(b)) in both WKY and SHR. Ang-(1-7) decreased MAP but had no significant effect on HR in SHR. Ang-(1-7) pretreatment inhibited the Ang II-induced elevation of blood pressure and HR in both WKY and SHR (Figure 1).

### 3.2. Vasoconstriction and Vasodilatation Function in WKY and SHR

**Figure 2:**
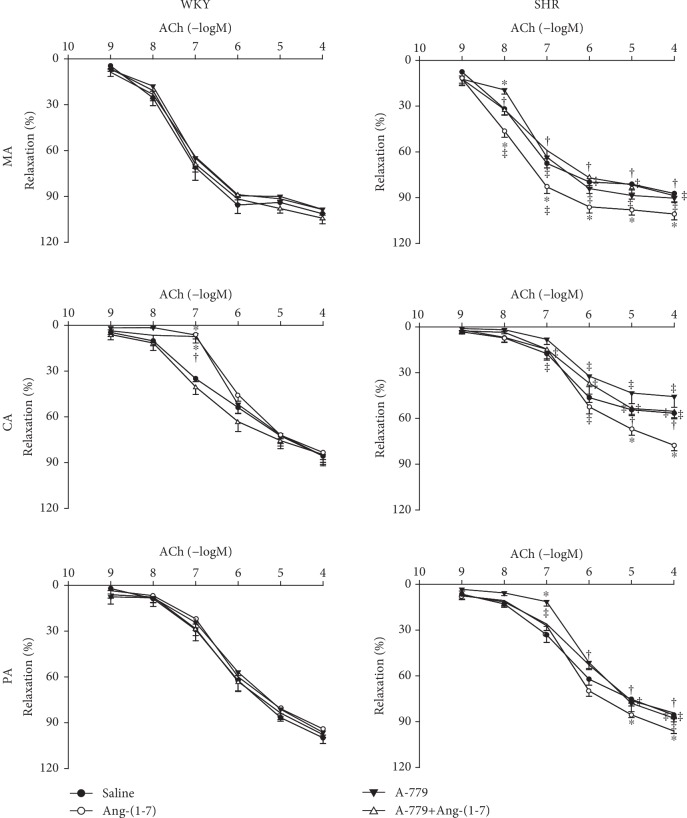
Effects of saline, Ang-(1-7) (10^−6^ mol/L), A-779 (10^−5^ mol/L), and A-779+Ang-(1-7) on ACh-induced dose-dependent relaxation in the MA, CA, and PA in WKY and SHR. Values are the mean ± SE. ^∗^*P* < 0.05 compared with saline. ^†^*P* < 0.05 compared with Ang-(1-7) alone. ^‡^*P* < 0.05 compared with WKY. *n* = 6 for each group.

There were no significant differences in body weight or HR between SHR and WKY, while the SBP and MAP of SHR were significantly higher than those observed in WKY (Table 1). The constriction of the MA, CA, and PA induced by high K^+^ solution was enhanced significantly in SHR (Table 2), while ACh-induced dose-dependent relaxations in the MA, CA, and PA were attenuated significantly in SHR compared with WKY, which was consistent with our previous finding [15] (Figure 2).

### 3.3. Effects of Ang-(1-7) and Ang II on Vascular Tension

Ang-(1-7) decreased basal vascular tension of the MA, CA, and PA in SHR, which was blocked by Mas receptor antagonist A-779. Ang II induced significant vasoconstriction in the MA, CA, and PA in both WKY and SHR models, which was blocked by the AT_1_ receptor antagonist losartan. Pretreatment with Ang-(1-7) on the arteries inhibited the Ang II-induced vasoconstriction in both WKY and SHR. Neither A-779 nor losartan had a significant effect on the basal vascular tension of the MA, CA, and PA in both WKY and SHR (Table 3).

### 3.4. Effects of Ang-(1-7) on ACh-Induced Vascular Relaxation

ACh-induced dose-dependent relaxation of the MA, CA, and PA was improved by treatment with Ang-(1-7) in SHR but not in WKY. This effect of Ang-(1-7) was blocked by A-779 pretreatment. A-779 had no significant effect on ACh-induced relaxations in both WKY and SHR (Figure 2).

### 3.5. Effects of Ang II on ACh-Induced Vascular Relaxation

**Figure 3:**
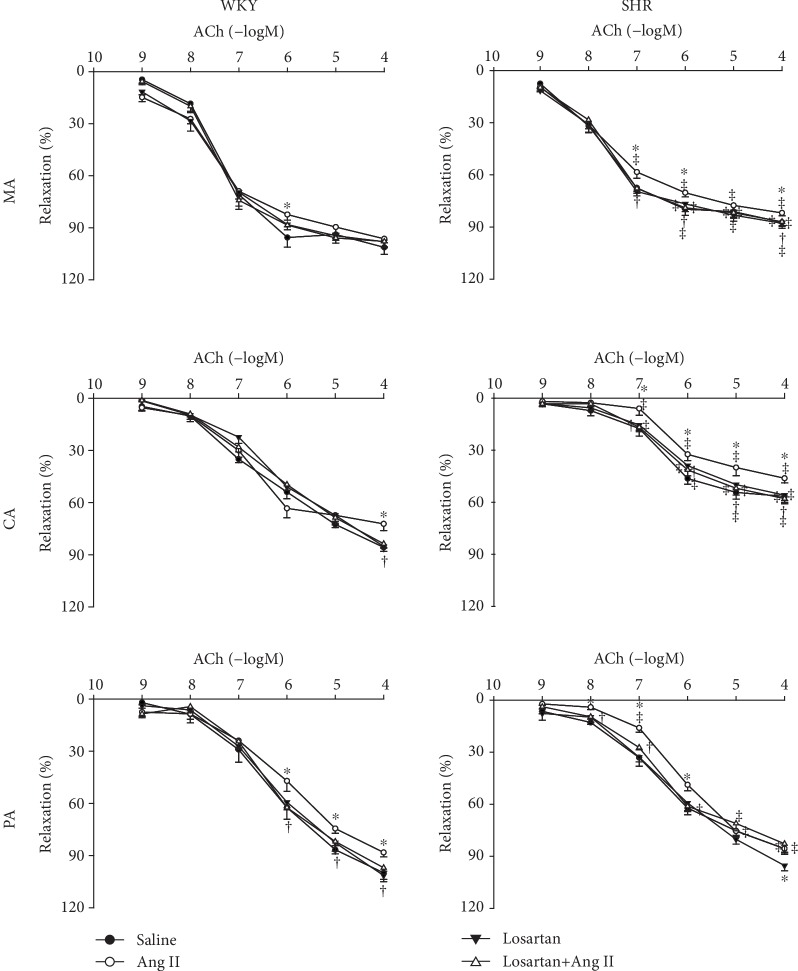
Effects of saline, Ang II (10^−6^ mol/L), losartan (10^−5^ mol/L), and losartan+Ang II on Ach-induced dose-dependent relaxation in the MA, CA, and PA in WKY and SHR. Values are the mean ± SE. ^∗^*P* < 0.05 compared with saline. ^†^*P* < 0.05 compared with Ang II alone. ^‡^*P* < 0.05 compared with WKY. *n* = 6 for each group.

Ang II attenuated ACh-induced relaxations in the MA, CA, and PA, and this phenomenon was more significant in SHR compared with WKY. Furthermore, this effect was blocked by the AT_1_ receptor antagonist losartan pretreatment. Losartan had no significant effect on ACh-induced relaxations in both WKY and SHR (Figure 3).

### 3.6. Effects of Ang-(1-7) on ACh-Induced Vascular Relaxation Response to Ang II

**Figure 4:**
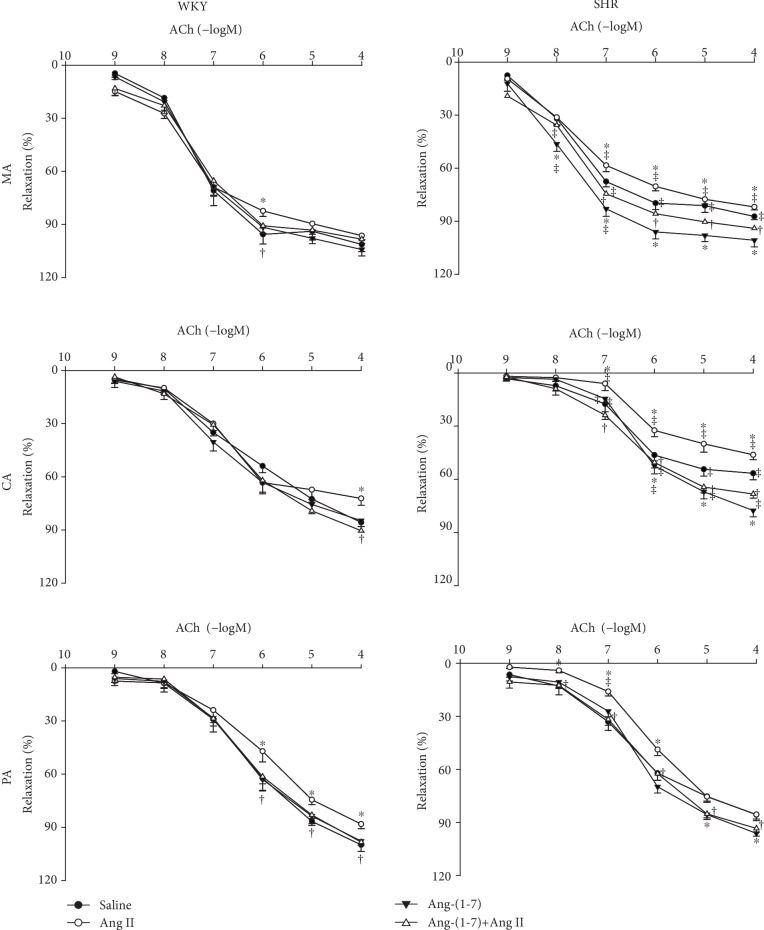
Effects of saline, Ang II (10^−6^ mol/L), Ang-(1-7) (10^−6^ mol/L), and Ang-(1-7)+Ang II on ACh-induced dose-dependent relaxation in the MA, CA, and PA in WKY and SHR. Values are the mean ± SE. ^∗^*P* < 0.05 compared with saline. ^†^*P* < 0.05 compared with Ang II alone. ^‡^*P* < 0.05 compared with WKY. *n* = 6 for each group.

Pretreatment with Ang-(1-7) significantly inhibited the effects of Ang II on ACh-induced vascular relaxation in the MA, CA, and PA in both WKY and SHR (Figure 4).

### 3.7. AT_1_ Receptor Protein Expression of Arteries

**Figure 5:**
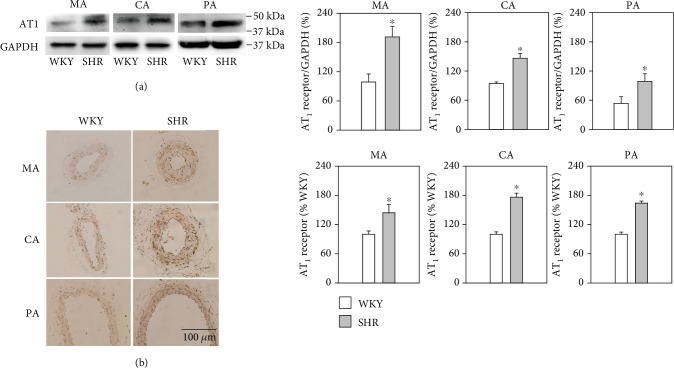
AT_1_ receptor protein expressions of the MA, CA, and PA in WKY and SHR detected by western blotting (a) and immunohistochemistry (b). Values are the mean ± SE. ^∗^*P* < 0.05 compared with WKY. *n* = 6 for each group.

Western blotting (Figure 5(a)) and immunohistochemistry (Figure 5(b)) revealed that AT_1_ receptor protein expressions in the MA, CA, and PA of SHR arteries were significantly higher than the expression levels measured in WKY arteries.

### 3.8. The NO, cGMP, and PKG Levels of MA, CA, and PA

**Figure 6:**
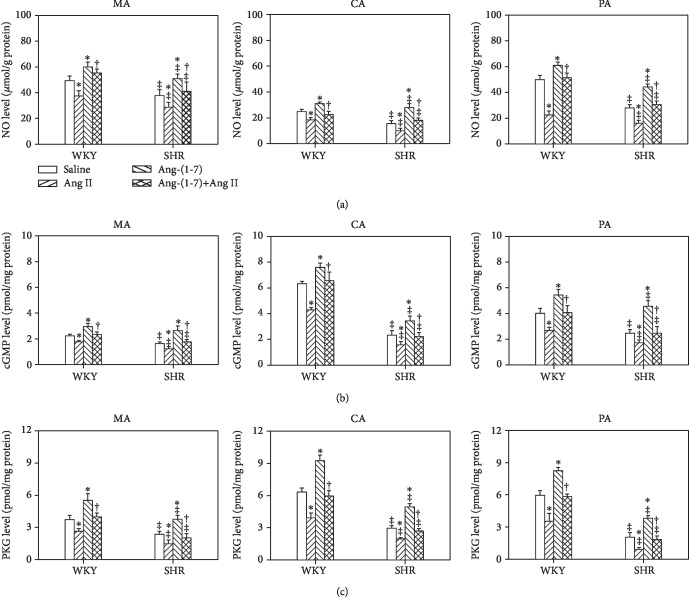
Effects of the saline, Ang II (10^−6^ mol/L), Ang-(1-7) (10^−6^ mol/L), and Ang-(1-7)+Ang II on the NO (a), cGMP (b), and PKG (c) levels of the MA, CA, and PA in WKY and SHR. Values are the mean ± SE. ^∗^*P* < 0.05 compared with saline. ^†^*P* < 0.05 compared with Ang II alone. ^‡^*P* < 0.05 compared with WKY. *n* = 6 for each group.

Compared with WKY, the NO, cGMP, and PKG levels of the MA, CA, and PA in SHR were decreased. Ang II further decreased, while Ang-(1-7) increased or normalized, NO, cGMP, and PKG levels of the MA, CA, and PA in SHR. In addition, the effect of Ang II on NO, cGMP, and PKG levels was inhibited by pretreatment with Ang-(1-7) on the arteries in both WKY and SHR (Figure 6).

## 4. Discussion

Endothelial dysfunction is a hallmark of hypertension and involved in further progression and deterioration. Our recent study has found that ACh-induced endothelium-dependent relaxation in the MA, CA, and PA of SHR was impaired significantly [15]. As we had talked about in a previous study [15], the MA, CA, and PA have been shown to play a major role in the damaging effects of hypertension; we therefore continue choosing these particular vessels for this study to investigate the effects of Ang (1-7) and Ang II on endothelial function. The present study demonstrates the following new findings: (1) Ang-(1-7) decreased blood pressure, while Ang II elevated it; (2) Ang-(1-7) enhanced ACh-induced vascular relaxation, while Ang II further attenuated it in the MA, CA, and PA of SHR. They were inhibited by pretreatment with Mas receptor antagonist A-779 and AT_1_ receptor antagonist losartan, respectively; (3) Ang-(1-7) decreased the basal tension of the MA, CA, and PA, while Ang II induced vasoconstriction in SHR; (4) pretreatment with Ang-(1-7) on the arteries inhibited the Ang II-induced pressor response, vasoconstriction, and the effects on ACh-induced relaxation in SHR; (5) compared with WKY, NO, cGMP, and PKG levels of the MA, CA, and PA were decreased, but the AT_1_ receptor expression of arteries was increased in SHR; (6) Ang II decreased but Ang-(1-7) increased NO, cGMP, and PKG levels of arteries; in addition, Ang-(1-7) pretreatment inhibited the Ang II-induced reduction of NO, cGMP, and PKG levels of arteries in SHR. These results indicate that activation of the Mas receptor by Ang-(1-7) restores endothelial dysfunction, decreases blood pressure, and inhibits the deteriorative effects of Ang II on vascular tension and Ang II induced pressor response through the NO-cGMP-PKG pathway.

In this study, SHR models had a significant increase in SBP and MAP as compared to WKY models. Additionally, when exposed to a high K^+^ solution to induce arterial constriction, SHR had a drastically enhanced response as compared to the WKY. SHR models also showed an impaired endothelium relaxation in their MA, CA, and PA after ACh induction. These results indicated that endothelial dysfunction of small arteries and consequential enhanced vasoconstriction and attenuated vasodilatation occurred in SHR.

Dense Mas receptors are found in the VECs [41] and VSMCs [42]. Ang-(1-7) is found playing important roles in the regulation of cardiovascular activity [43, 44], and more interestingly, the regulation roles of Ang-(1-7) are different in different sites of the body. Our recent studies have found that microinjection of Ang-(1-7) into either the paraventricular nucleus (PVN) or the rostral ventrolateral medulla (RVLM) increases renal sympathetic nerve activity and MAP in renovascular hypertensive rats [39, 40, 45]. Ang-(1-7) in the PVN or RVLM increases NAD(P)H oxidase activity and superoxide anion level, and superoxide anions but not NO modulate the effects of Ang-(1-7) in the PVN or RVLM on sympathetic activity and blood pressure in normal rats or chronic heart failure rats [46, 47]. However, in regard to cardiovascular activity, Ang-(1-7) plays opposite regulating roles in peripheral tissues. Ang-(1-7) has been shown to decrease blood pressure [48] and induce mesenteric arterial relaxation in normotensive rat models [49, 50]. Ang-(1-7) has also been shown to improve endothelial function and delay the development of cardiac remodeling, even heart failure, in rats with myocardial infarction [51]. In the present study, we found that Ang-(1-7) enhanced ACh-induced vascular relaxation and decreased blood pressure and the basal tension of the MA, CA, and PA in SHR, which were blocked by pretreatment with Mas receptor antagonist A-779. These findings indicated that Ang-(1-7) has a beneficial effect to improve endothelial function and decrease blood pressure and vascular tension by activation of the Mas receptor in the arteries in a hypertensive state. In addition, a study has reported that Ang-(1-7)-dependent vasorelaxation of the renal artery was sensitive to antagonists against not only Mas but also AT_1_, AT_2_, and bradykinin receptor subtypes [52], which we will study in the future.

It has been reported that the Ang-(1-7)/Mas receptor axis presumably acts as a counterregulator of classic Ang II/AT_1_-mediated effects in heart failure [53], emotional stress [54], or inflammatory conditions [55]. Ang-(1-7) plays an antagonistic role in the Ang II-induced pressor effect [34] and inhibits Ang II-induced phosphorylation of ERK, p38, and JNK [35]. Activation of the Mas receptor blocks Ang II-induced vasoconstriction [33]. In the present study, we found that Ang II elevated blood pressure significantly, attenuated ACh-induced vascular relaxation, and induced the constriction of the MA, CA, and PA in SHR, which were inhibited by pretreatment with the AT_1_ receptor antagonist losartan, suggesting the worse effects of activity of the Ang II/AT_1_ receptor on endothelial dysfunction and hypertension. It is important to note that pretreatment with Ang-(1-7) on the arteries inhibited the Ang II-induced pressor response and vasoconstriction, as well as the effects on ACh-induced vascular relaxation in SHR. The results indicate that Ang-(1-7) not only improves the impaired endothelial function by activation of the Mas receptor directly but also protects endothelial function through antagonizing the adverse effects of Ang II indirectly, which emphasized the important roles of Ang-(1-7) in restoring endothelial dysfunction in hypertension.

In addition, we previously found that the Mas receptor protein expressions in the MA, CA, and PA of SHR were decreased [15], while in this study, AT_1_ receptor protein expressions of the arteries of SHR were increased significantly compared with WKY. From these results, we speculated that the activity of the Ang-(1-7)/Mas receptor was impaired and the activity of Ang II/AT_1_ was enhanced in SHR, which might be an important reason for inducing endothelial dysfunction and subsequent enhanced vasoconstriction and attenuated vasodilatation in a hypertensive state. Improvement of the activity of the Ang-(1-7)/Mas receptor would be beneficial for inhibition of the development and progression of endothelial dysfunction in hypertension.

Some studies have shown that NO mediates effects of Ang-(1-7) in peripheral tissues. Ang-(1-7) induces canine middle cerebral artery relaxation through stimulating NO release from endothelial cells [56]. Ang-(1-7) activates eNOS and increases the production of NO in human aortic endothelial cell, Mas-transfected Chinese hamster ovary cells [57], and cardiomyocytes [58]. As we know, NO released from endothelial cells induces VSMC relaxation through activation of the intracellular cGMP-PKG signal pathway. The present study found that NO, cGMP, and PKG levels of the MA, CA, and PA in SHR were much lower than that in WKY, which further suggests the presence of endothelial dysfunction in SHR and that the NO-cGMP-PKG signal pathway plays important roles during endothelial dysfunction of SHR. Ang-(1-7) increased or normalized, while Ang II further decreased, NO, cGMP, and PKG levels of the MA, CA, and PA in SHR. More importantly, pretreatment with Ang-(1-7) on the arteries inhibited the Ang II-induced reduction of NO, cGMP, and PKG levels in SHR. These results suggest that the NO-cGMP-PKG signal pathway might be an important intracellular signaling mechanism mediating the effects of Ang-(1-7) or Ang II on vascular endothelial function. Activation of Mas receptors on the endothelial cell membrane by Ang-(1-7) might stimulate NO generation and then NO relaxed the VSMCs through the cGMP-PKG signal pathway, while activation of AT_1_ receptors on the endothelial cell membrane by Ang II decreased NO generation and subsequent activation of the cGMP-PKG signal pathway. As to the mechanisms of the inhibitory influence of Ang-(1-7) on Ang II effects, we speculated that it might be some molecule mechanisms of activation or inactivation of eNOS inside of the endothelial cell, which need to be studied in the future.

In conclusion, the activity of the Ang-(1-7)/Mas receptor in the arteries was impaired and the activity of the Ang II/AT_1_ receptor was enhanced in SHR. Activation of the Mas receptor by Ang-(1-7) decreased blood pressure, improves endothelial function, and inhibits the deteriorative effects of Ang II on endothelial function, vascular tension, and hypertension through the NO-cGMP-PKG pathway in SHR. Improvement of Ang-(1-7)/Mas receptor activity in the arteries might be developed as a therapeutic strategy to ameliorate endothelial dysfunction and inhibition of hypertension.

## Figures and Tables

**Table 1:** Body weight, SBP, MAP, and HR in one representative group of WKY and SHR.

	WKY	SHR
Body weight (g)	322.4 ± 4.7	320.5 ± 3.9
SBP (mm Hg)	114.7 ± 4.8	195.1 ± 4.9^∗^
MAP (mm Hg)	89.6 ± 4.2	135.4 ± 4.2^∗^
HR (beats/min)	384.3 ± 11.3	379.4 ± 12.6

SBP: systolic blood pressure; MAP: mean arterial pressure; HR: heart rate. Values are expressed as the mean ± SE. ^∗^*P* < 0.05 compared with the WKY rats. *n* = 6 for each group.

**Table 2:** The high K^+^-induced contraction (mg/mm) in MA, CA, and PA in WKY and SHR.

	WKY	SHR
MA	428.5 ± 52.9	876.9 ± 60.6^∗^
CA	100.31 ± 12.3	202.5 ± 18.6^∗^
PA	115.4 ± 14.3	181.0 ± 12.4^∗^

Values are the mean ± SE. ^∗^*P* < 0.05 compared with WKY. *n* = 6 for each group.

**Table 3:** Influence of saline, Ang-(1-7), Ang II, Ang-(1-7)+Ang II, A-779, A-779+Ang-(1-7), losartan, and losartan+Ang II on the basal vascular tension (mg/mm) in WKY and SHR.

		Saline	Ang-(1-7)	Ang II	Ang-(1-7)+Ang II	A-779	A-779+Ang-(1-7)	Losartan	Losartan+Ang II
WKY	MA	-0.9 ± 3.0	-10.2 ± 2.4^∗^	155.2 ± 16.3^∗^	6.3 ± 3.0^‡^	6.3 ± 3.6	5.6 ± 2.8^#^	0.1 ± 1.1	6.2 ± 1.1^‡^
	CA	0.5 ± 2.7	-2.9 ± 2.5	46.5 ± 7.9^∗^	13.2 ± 2.3^∗^^‡^	4.6 ± 2.0	5.4 ± 2.0	0.1 ± 1.2	-1.1 ± 2.8^‡^
	PA	3.3 ± 1.9	-0.6 ± 3.1	201.0 ± 14.1^∗^	19.4 ± 4.7^∗^^‡^	8.1 ± 3.2	3.4 ± 2.3	5.3 ± 2.3	12.2 ± 3.0^∗^^‡^

SHR	MA	2.7 ± 2.4	-15.7 ± 2.5^∗^	137.4 ± 18.3^∗^	5.6 ± 3.2^‡^	8.6 ± 2.5	4.2 ± 2.9^#^	1.4 ± 3.9	-0.7 ± 3.7^‡^
	CA	3.0 ± 1.9	-9.1 ± 1.7^∗^^†^	76.3 ± 7.4^∗^^†^	15.8 ± 3.5^∗^^‡^	3.0 ± 1.5	4.1 ± 3.0^#^	-2.3 ± 2.1	8.1 ± 2.1^‡^
	PA	-2.1 ± 2.0	-12.4 ± 3.0^∗^^†^	230.6 ± 17.5^∗^	13.5 ± 4.6^∗^^‡^	3.5 ± 3.4	5.4 ± 2.0^#^	1.0 ± 1.7	3.0 ± 3.5^‡^

Data showed the changes (mg/mm) of vascular tension from the values before chemical intervention. Values are expressed as the mean ± SE. ^∗^*P* < 0.05 vs. the saline. ^†^*P* < 0.05 vs. WKY. ^‡^*P* < 0.05 compared with Ang II alone. ^#^*P* < 0.05 compared with Ang-(1-7) alone. *n* = 6 for each group.

## Data Availability

All data supporting the findings of this study are available within the main manuscript or from the corresponding authors upon reasonable request.
